# Diagnostic value of dual-energy CT virtual monochromatic imaging for supraspinatus tendon injuries: a comparison with standard CT and MRI

**DOI:** 10.1007/s00330-025-11760-5

**Published:** 2025-06-14

**Authors:** Suwei Liu, Qizheng Wang, Yali Li, Ming Ni, Chenyu Jiang, Wenhuan Li, Huishu Yuan

**Affiliations:** 1 Department of Radiology, Peking University Third Hospital, Beijing, China; 2 CT Research Center, GE Healthcare China, Beijing, China

**Keywords:** Dual-energy scanned projection, Magnetic resonance imaging, Shoulder pain, Supraspinatus, Diagnosis

## Abstract

**Objectives:**

This study aimed to compare the diagnostic value of dual-energy computed tomography (DECT) virtual monochromatic imaging with that of standard computed tomography (SCT) in evaluating supraspinatus tendon injuries.

**Materials and methods:**

This retrospective study involved patients who underwent a single-source DECT system, 3.0-T MRI, and shoulder arthroscopy within 14 days. Three radiologists independently and randomly evaluated SCT, mono+ 50 keV, mono+ 90 keV, and MRI images to detect supraspinatus tendon tears and recorded their diagnostic confidence. Regions of interest were delineated to measure the CT attenuation values of torn, degenerated, and normal tendon regions in SCT, mono+ 50 keV, and mono+ 90 keV images.

**Results:**

A total of 100 patients with supraspinatus tendon injuries were included. Significant differences in detecting supraspinatus tendon injuries were observed between SCT and DECT (*p* < 0.05) and between SCT and MRI (*p* < 0.05), without showing differences in detecting supraspinatus tears between DECT and MRI. CT attenuation values of tears in SCT, mono+ 50 keV, and mono+ 90 keV images were significantly lower than those of degenerative and normal regions (*p* < 0.001). Optimal CT attenuation values to differentiate between tears and degeneration on SCT, mono+ 50 Kev, and mono+ 90 Kev were 17.4, 28.0, and 14.2 with area under the curve (AUC) of 0.920, 0.978, and 0.938, respectively. Additionally, these values between degeneration and normal regions were 23.8 HU, 36.1 HU, and 19.6 HU with AUCs of 0.967, 0.970, and 0.946, respectively.

**Conclusion:**

DECT demonstrated high diagnostic accuracy and reliability for qualitative and quantitative assessment of supraspinatus tendon injuries.

**Key Points:**

***Question*** *Is DECT virtual monochromatic imaging capable of diagnosing supraspinatus tendon injuries when MRI is unavailable or contraindicated*?

***Findings*** *DECT demonstrated high diagnostic accuracy and reliability for qualitative and quantitative assessment of supraspinatus tendon injuries*.

***Clinical relevance*** *DECT can effectively and reliably diagnose supraspinatus tendon tears using both qualitative and quantitative methods and holds promise as a supplementary tool for MRI*.

**Graphical Abstract:**

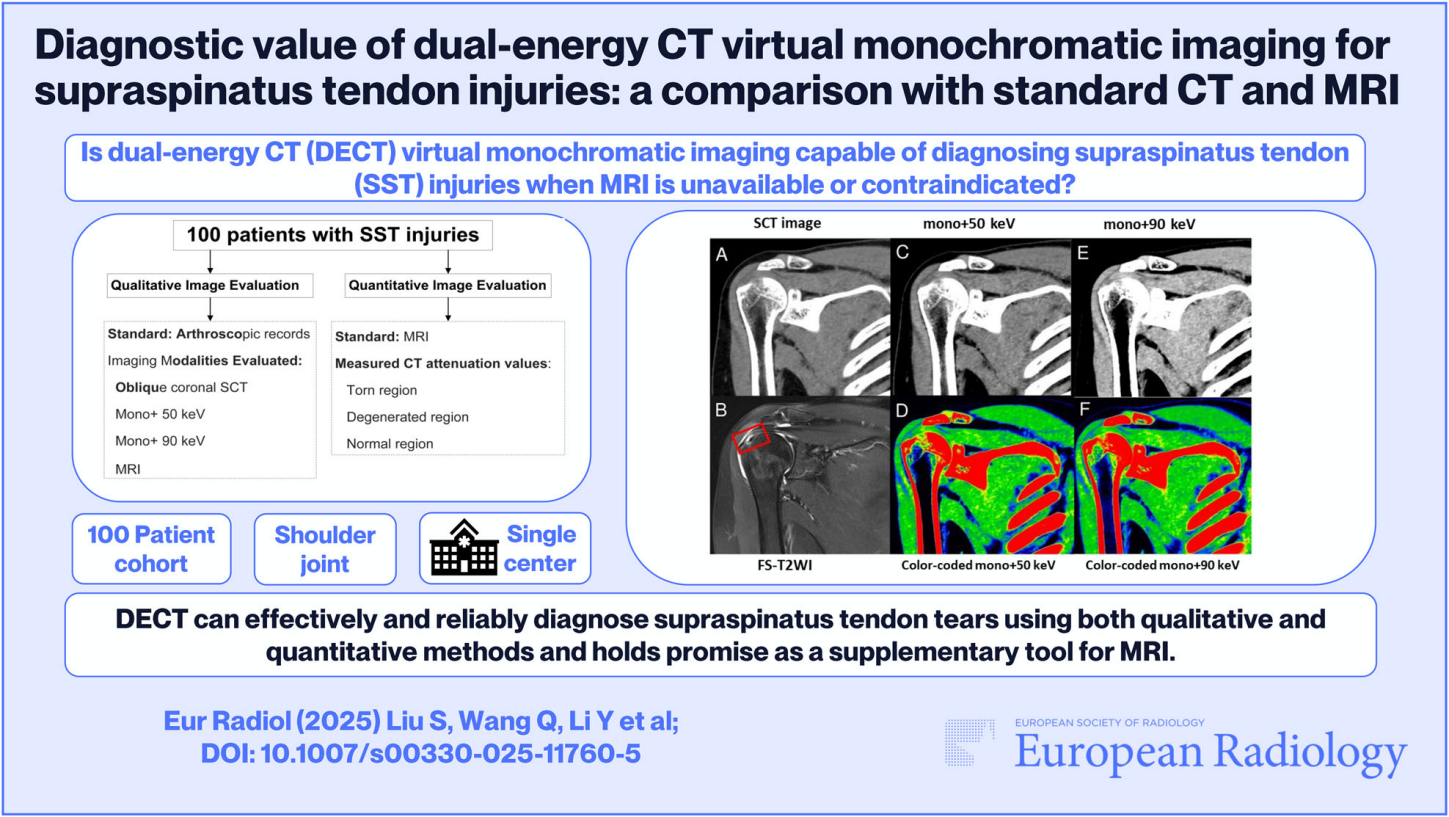

## Introduction

Rotator cuff injuries are a common cause of shoulder pain and restricted shoulder joint movement, with the supraspinatus tendon being the most frequently injured. These injuries typically result from impingements, degenerative changes, subacromial bone spurring, poor blood supply, or overuse [1]. Accurate and timely diagnosis of rotator cuff injuries can alleviate pain, reduce disability rates, and lower treatment costs [2].

In clinical settings, physical examination is the standard approach to evaluating rotator cuff injuries [3]. However, because the symptoms overlap with other conditions, such as shoulder instability and impingement, determining the exact cause of shoulder pain through physical examination alone is challenging. MRI remains the gold standard for diagnosing rotator cuff injuries [3]. However, MRI has limitations for patients with acute trauma or contraindications such as magnetic implants, pacemakers, claustrophobia, and obesity [4]. In such cases, ultrasound is often the imaging modality of choice due to its convenience, affordability, and ability to provide real-time, dynamic assessment. However, its limitations include operator dependence, reduced ability to visualize deeper structures, and decreased accuracy in patients with obesity or limited shoulder mobility. Therefore, a supplementary imaging modality is required to address these limitations when diagnosing rotator cuff injuries.

Standard computed tomography (SCT) enhances the contrast resolution of injured areas and surrounding structures by adjusting the window width and level using three-dimensional (3D) imaging technology. However, limited soft tissue attenuation contrast diminishes the visibility of the tendons and ligaments [5]. Thus, SCT is primarily used to diagnose bone injuries and is rarely applied to the clinical evaluation of soft tissues. CT arthrograms are an alternative for evaluating the rotator cuff, though it is often invasive and inconvenient.

Dual-energy computed tomography (DECT) leverages attenuation differences at various energy levels to distinguish between soft tissue joint structures. Likewise, virtual monoenergetic imaging (VMI) holds significant clinical value [5, 6]. In a study involving patients with brain tumors undergoing non-contrast-enhanced cranial CT, Tanoue et al [7] found that the tumor contrast and diagnostic confidence of DECT-VMI at 40 keV surpassed those of SCT images. Similarly, Liu et al [8] demonstrated that DECT is effective in diagnosing anterior cruciate ligament (ACL) tears using both qualitative and quantitative approaches, showing great potential for assessing the integrity of injured ACLs.

Research on the use of DECT to assess supraspinatus tendon injuries is limited. Therefore, this study aimed to assess the qualitative and quantitative value of DECT-VMI for diagnosing supraspinatus tendon injuries, hypothesizing that DECT-VMI is comparable to MRI in terms of accuracy and reliability. These findings may provide a cost-effective alternative in patients with contraindications to MRI, facilitating early detection of injuries and offering clinical evidence of bone injury.

## Materials and methods

### Patient selection

**Fig. 1:**
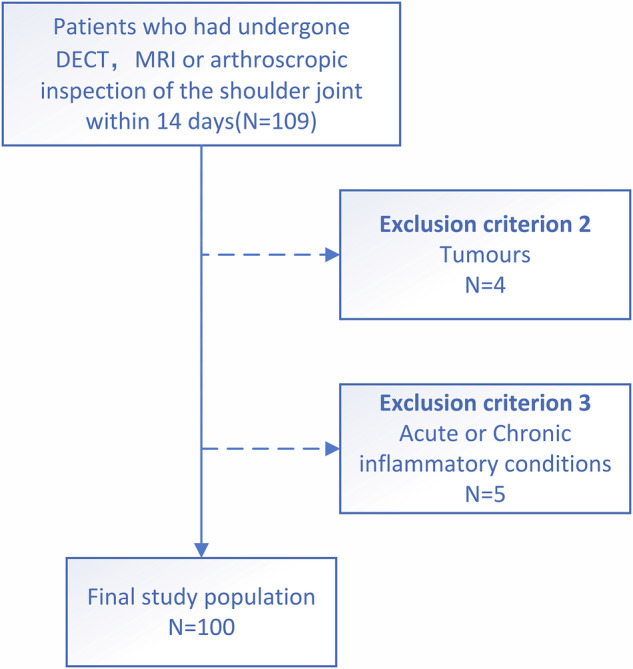
Flowchart of patient inclusion

This retrospective diagnostic study was approved by the Institutional Review Board (M2024188). It included patients who underwent arthroscopic surgery for rotator cuff injuries and preoperative DECT and MRI between November 2023 and April 2024. The inclusion criteria were: (1) age between 14 years and 80 years; (2) the interval between DECT, MRI, and arthroscopic surgery was within 14 days; and (3) first-time surgery with comprehensive surgical records. The exclusion criteria were: (1) presence of tumors; and (2) acute or chronic shoulder arthritis, including osteoarthritis or inflammatory arthritis (Fig. 1). We recorded patients’ baseline information, including age, sex, injured side, tear type, and scope of arthroscopic tear—small tears, < 1 cm; medium tears, between 1 cm and 3 cm; large tears, between 3 cm and 5 cm; and huge tears, > 5 cm.

### CT protocol

CT was performed using a single-source DECT system (Revolution ES, GE Medical Systems). Acquisition parameters were as follows: GSI KV mode with rapid switching between 80–140 kVp; tube current of 190 mA, collimation thickness of 0.625 mm, helical scan mode, rotation time of 0.8 s, pitch of 0.984, pixel size of 0.5 × 0.5, 512 × 512 matrix, and field of view (FOV) of 250 mm. The CT dose index volume was 10.21 mGy, with a dose-length product of approximately 320 mGy·cm for a scan length of 32 cm. All participants wore lead-protective vests to protect against scattered radiation.

### MRI protocol

MRI was performed using a 3-T system (MAGNETOM Lumina, Siemens Healthineers). The protocol included a T1-weighted fast spin-echo sequence without fat suppression (oblique coronal plane) and proton density-weighted sequences with fat suppression (axial, oblique sagittal, and oblique coronal planes) with a 3-mm slice thickness. Identical pulse sequence parameters (echo time, repetition time, and flip angle), FOV, and acquisition matrix were maintained across all examinations, as detailed in Table S1.

### CT postprocessing

The image data (datafile) was reconstructed by a radiologist using a GE Advanced Workstation (ADW4.7, GE Medical Systems). The reconstruction included coronal, sagittal, and axial oblique images.

### Image analysis

#### Determining the optimal VMI and slice thickness

Optimal image quality and lesion visibility were determined by selecting the most suitable VMI. Images ranging between 40 keV and 140 keV at integer energy levels, combined with slice thicknesses of 1 mm, 2 mm, and 3 mm, resulted in 11 × 3 sets of images evaluated in ten patients. Two experienced musculoskeletal radiologists (with 20 years and 35 years of experience) evaluated and compared these images based on the image noise, lesion prominence, image quality, and diagnostic confidence to identify the optimal VMI and slice thickness. The process for determining the optimal VMI and slice thickness is explained in Table S2.

### Qualitative analysis of selected images

After weighing the image quality against lesion prominence, mono+ 50 keV and mono+ 90 keV images were ultimately selected as the best ones, with a 3-mm slice thickness and 1 mm interval. The mono+ 50 keV color-code image was set to a window width of 600–800 and a window level of 40–70. The mono+ 90 keV color-code image’s window width was set to 400–500, and the window level to 30–60.

**Fig. 2:**
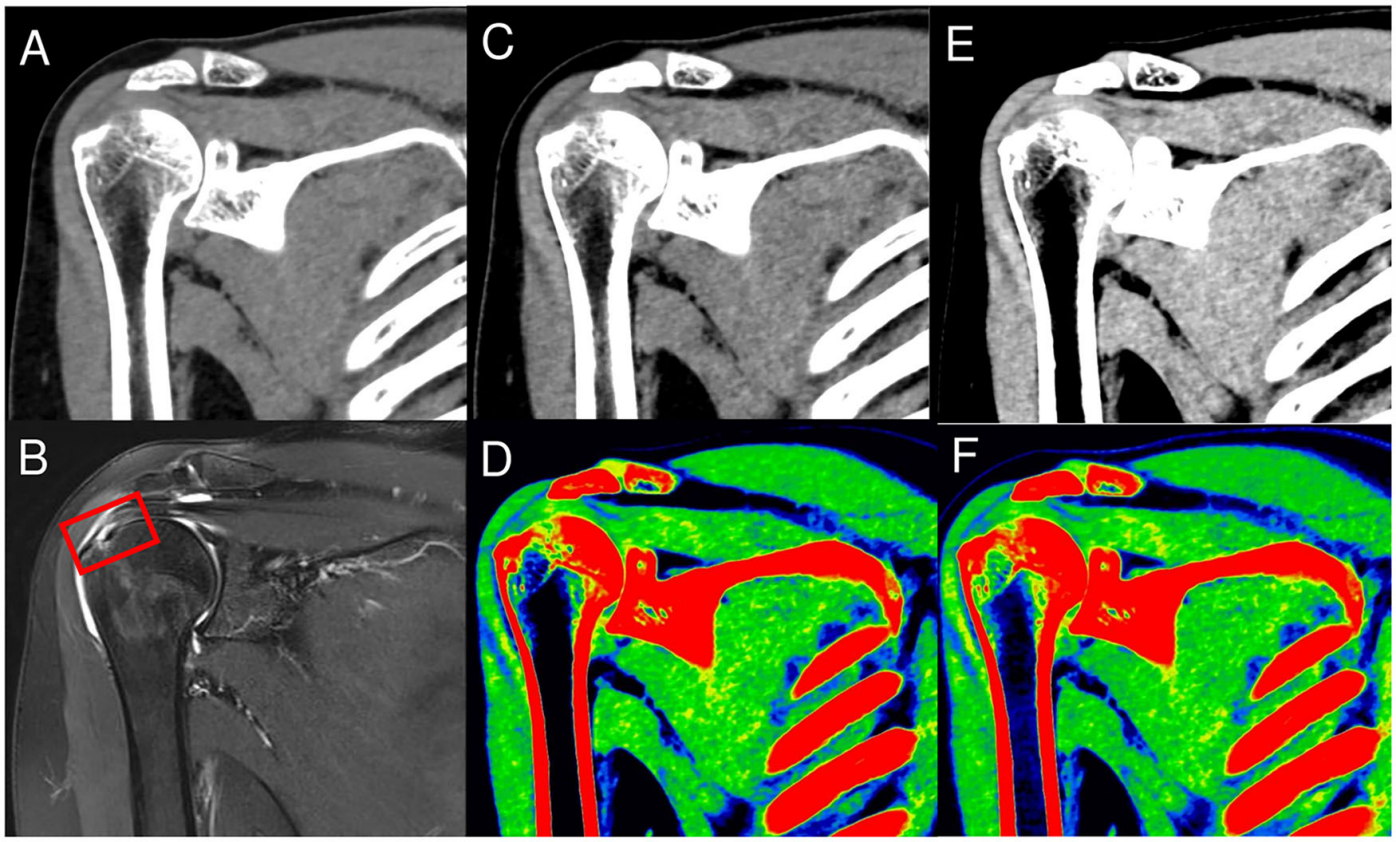
Oblique coronal plane images of a 52-year-old man with right supraspinatus tendon tear: (**A**) SCT image, (**B**) proton density weighted fat-saturated MRI image showing a tear in the supraspinatus tendon (red dotted rectangle), (**C**) DECT images with mono+ 50 keV, (**D**) color-coded DECT images with mono+ 50 keV, (**E**) DECT images with mono+ 90 keV, and (**F**) color-coded DECT images with mono+ 90 keV. SCT, standard computed tomography; DECT, dual-energy computed tomography; MRI, magnetic resonance imaging

**Fig. 3:**
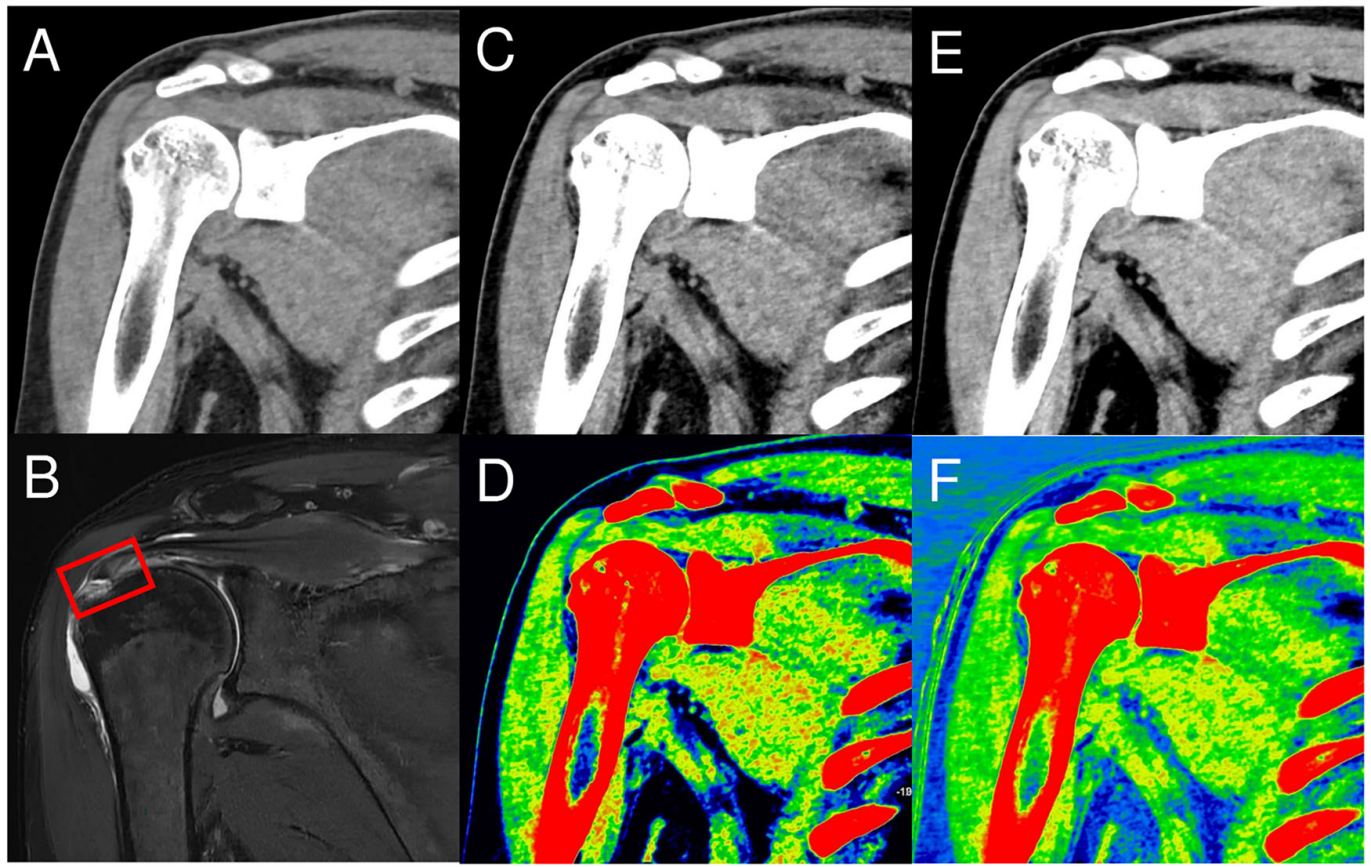
Oblique coronal plane images of a 49-year-old man with right supraspinatus tendon tear: (**A**) SCT image, (**B**) proton density weighted fat-saturated MRI image showing a tear in the supraspinatus tendon (red dotted rectangle), (**C**) DECT images with mono+ 50 keV, (**D**) color-coded DECT images with mono+ 50 keV, (**E**) DECT images with mono+ 90 keV, and (**F**) color-coded DECT images with mono+ 90 keV. SCT, standard computed tomography; DECT, dual-energy computed tomography; MRI, magnetic resonance imaging

Considering arthroscopic surgical records as the gold standard, in which both partial and full thickness tears, arthroscopically confirmed, were considered tears, three musculoskeletal radiologists with varying experience levels (20 years, 12 years, and 5 years) independently evaluated oblique coronal SCT, mono+ 50 keV, mono+ 90 keV, and MRI image sets to diagnose supraspinatus tendon tears using a GE workstation (Figs. 2 and 3). In addition, diagnostic confidence scores were recorded for uncolored and color-coded images. All patient image information was anonymized, and the readers were blinded to the demographic and clinical data, including name, sex, age, and presence of supraspinatus tendon injuries, and each radiologist repeated the evaluation after 2 weeks. We rated their diagnostic confidence in detecting supraspinatus tendon tears using a five-point Likert scale as follows: 1 = very poor, 2 = barely acceptable, 3 = acceptable, 4 = good, and 5 = very good.

### Quantitative analysis of selected images

**Fig. 4:**
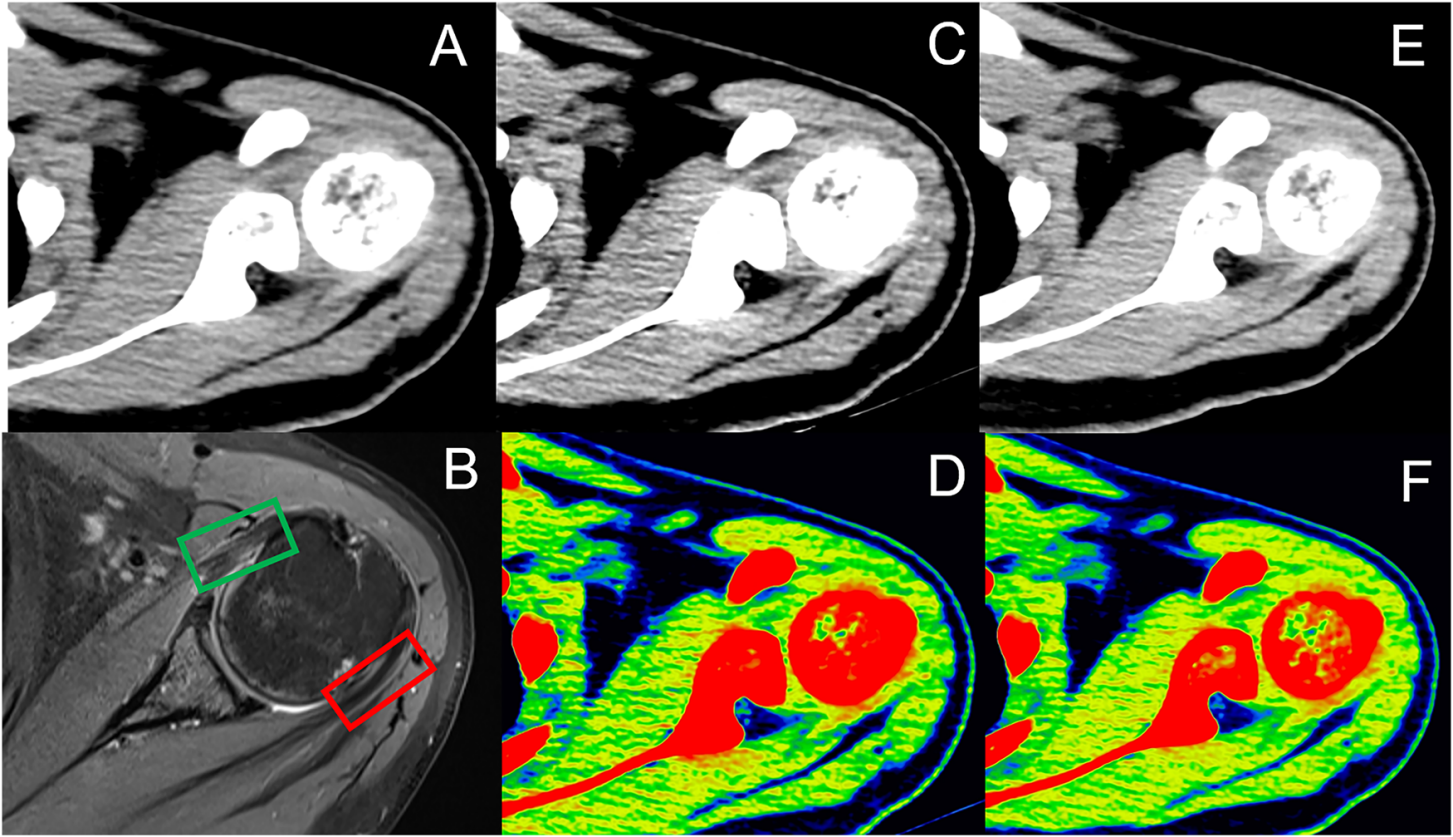
Transverse plane images of a 46-year-old woman with normal left infraspinatus tendon and degenerative subscapularis tendon: (**A**) SCT image, (**B**) proton density weighted fat-saturated MRI image displaying a normal infraspinatus tendon (red rectangular box) and a degenerated subscapularis tendon (green rectangular box), (**C**) DECT images with mono+ 50 keV, (**D**) color-coded DECT images with mono+ 50 keV, (**E**) DECT images with mono+ 90 keV, and (**F**) color-coded DECT images with mono+ mode90 keV. SCT, standard computed tomography; DECT, dual-energy computed tomography; MRI, magnetic resonance imaging

The tear areas were described as an arthroscopically visible rupture of the tendon fibers, which can be identified with increased signal intensity on T2/PDWI and a clear discontinuity of the tendon structure. Degenerative areas were characterized by tendinosis and arthroscopic tendon integrity, which appear as a thickened and heterogeneous tendon with increased signal intensity on T2/PDWI without a full-thickness tear. Normal areas were defined by arthroscopic tendon integrity and homogeneous low signal intensity on both T1/T2WI, indicating intact and healthy tendon fibers without any signs of abnormality. The diagnostic MRI findings of an experienced radiologist (H.Y., with 35 years of experience) were used as a reference standard. Three musculoskeletal radiologists (with 20 years, 12 years, and 5 years of experience) reconstructed oblique coronal and axial CT images at the GE workstation using this as a reference standard. In the SCT, mono+ 50 keV, and mono+ 90 keV images, elliptical regions of interest of approximately 1.0 cm² were selected to measure the CT attenuation values of the torn, degeneration, and normal regions (Fig. 4). Each region was measured three times with a 2-week interval between measurements.

Prior to image evaluation, all readers underwent training to ensure adherence to the assessment standards. Images from 10 individuals not involved in this study were used in the training, which included detailed instructions on CT image reconstruction methods, sample images, and scoring definitions. Each reader selected their preferred window level, magnification, and scrolling method.

### Statistical analysis

Statistical analysis was performed using a dedicated commercial software (MedCalc for Windows, Version 20.022, MedCalc). Analysis was conducted after categorizing the lesions as either “Tear” (0) or “No Tear” (1) for SCT, DECT, and MRI images. Findings were organized into cross tables, and diagnostic accuracy parameters (sensitivity, specificity, positive predictive value [PPV], negative predictive value [NPV], and area under the curve [AUC]) were calculated to assess the ability to detect supraspinatus tendon injuries. The Friedman test analyzed the radiologists’ diagnostic confidence across the three image types, whereas the Kendall rank correlation coefficient examined the relationship between tear size and SCT/DECT detection results. The Kruskal–Wallis *H*-test was used to compare CT attenuation values among torn, degenerated, and normal tendons in SCT, mono+ 50 keV, and mono+ 90 keV images. Receiver operating characteristic curve analysis was then performed to calculate the sensitivity, specificity, and accuracy. Intrarater and interrater consistencies for the three image measurements were analyzed using the intraclass correlation coefficient.

## Results

### Patient characteristics

The characteristics of the 100 patients included in this study are summarized in Table 1. Among these patients, 65 had supraspinatus tendon tears (23 males/42 females; 15 left/50 right shoulders; mean age: 60.0 ± 9.4 years, range: 36–79 years), comprising 16 partial tears and 49 full-thickness tears. The tears were categorized into four groups as follows: small (34.7%), medium (38.8%), large (14.3%), and huge (12.2%). Meanwhile, 35 had no supraspinatus tendon tears (23 males/12 females; 14 left/21 right shoulders; mean age: 48.2 ± 13.3, range: 14–75 years).

**Table 1:** Patient characteristics

	STT (*n* = 65)	No STT (*n* = 35)
Age (year)	60.0 ± 9.4 (36–79)	48.2 ± 13.3 (14–75)
Sex (*n*)		
Male	23 (35.4%)	23 (65.7%)
Female	42 (64.6)	12 (34.3%)
Injured side (*n*)		
Left	15 (23.1%)	14 (40.0%)
Right	50 (76.9%)	21 (60.0%)
Tear type (*n*)		
Partial tear	16 (24.6%)	
Full-thickness tear	49 (75.4%)	
Scope of tear (*n*)		
Small tears	17 (34.7%)	NA
Medium tears	19 (38.8%)	NA
Large tears	7 (14.3%)	NA
Huge tears	6 (12.2%)	NA

*STT* supraspinatus tendon tear, *NA* not available

### Diagnostic performance of DECT in detecting supraspinatus tendon injuries

**Fig. 5:**
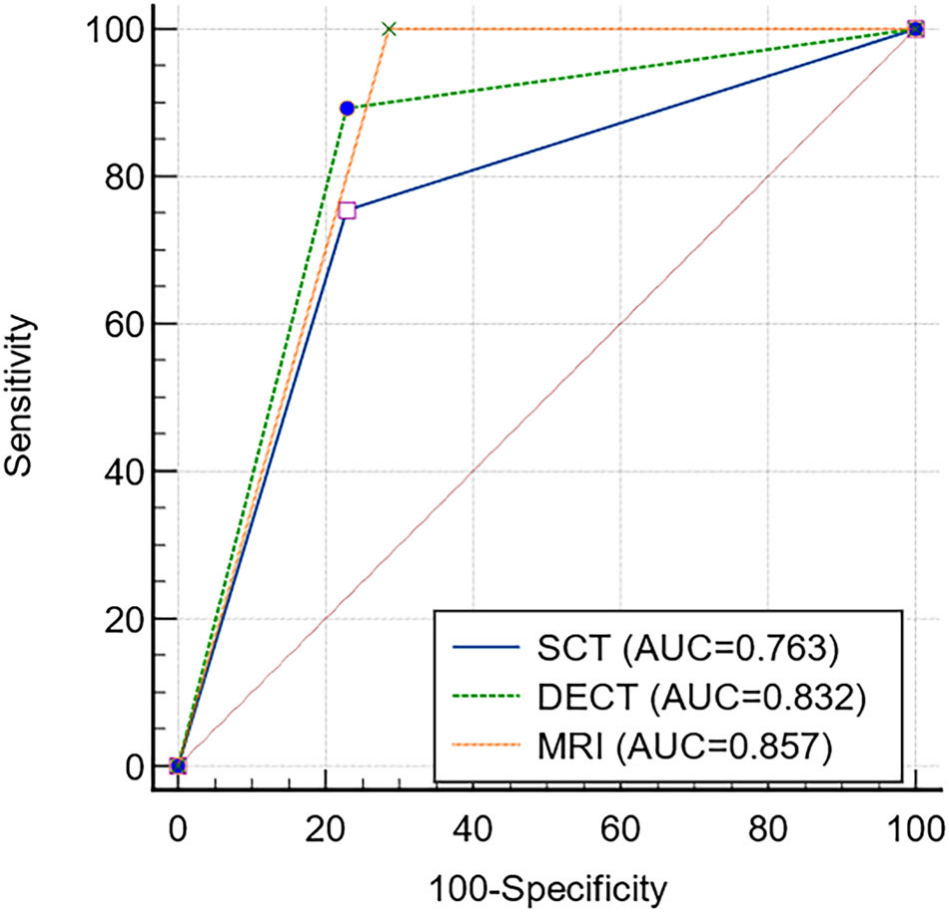
Diagnostic performance of the supraspinatus tendon injury SCT, DECT, and MRI. The AUCs were 0.763, 0.832, and 0.857 for CT, DECT, and MRI, respectively. SCT, standard computed tomography; DECT, dual-energy computed tomography; MRI, magnetic resonance imaging; AUC, area under the curve

Table 2 presents the validity and reliability of SCT, DECT, and MRI in diagnosing supraspinatus tendon injuries. Arthroscopy revealed 65 supraspinatus tears and 35 intact tendons. SCT identified 57 tears and 43 intact tendons; DECT identified 66 tears and 34 intact tendons; and MRI detected 75 tears and 25 intact tendons. Specifically, SCT and DECT showed 16 false negatives (16 out of 43 negative cases) and 7 false negatives (7 out of 34 negative cases), respectively. In contrast, the MRI modality showed no false negatives: 0 out of 25 negative cases. The accuracy rates were 76.0% for SCT, 85.0% for DECT, and 90.0% for MRI. Sensitivity was 75.4% for SCT, 89.2% for DECT, and 100% for MRI. Specificity was 77.1% for SCT, 77.1% for DECT, and 71.4% for MRI. The AUCs were 76.3% for SCT, 83.2% for DECT, and 85.7% for MRI (Fig. 5 and Table 2). The DeLong test indicated significant differences between SCT and DECT/MRI images in detecting supraspinatus tendon injuries (*p* < 0.01) without significant difference between MRI and DECT images (*p* > 0.05, *p* = 0.46). A strong correlation was identified between the scope of arthroscopic tear and diagnostic outcomes for SCT and DECT (τ = 0.678/0.701).

**Table 2:** The diagnostic performance of the supraspinatus tendon injury, SCT, DECT, and MRI

Inspection methods	Accuracy	Sensitivity	Specificity	PPV	NPV	AUC
SCT(%)^†^	76.0 [66.4–84.0] (76/100)	75.4 [67.2–82.1] (49/65)	77.1 [66.4–84.0] (27/35)	86.0 [74.2–93.4] (49/57)	62.8 [46.7–77.0] (27/43)	76.3 [67.5–85.1]
DECT(%)^†^	85.0 [76.5–91.4] (85/100)	89.2 [81.0–94.1] (58/65)	77.1 [63.3–86.9] (27/35)	87.9 [77.5–94.6] (58/66)	79.4 [62.1–91.3] (27/34)	83.2 [75.2–91.2]
MRI(%)^†^	90.0 [82.4–95.1] (90/100)	100.0 [94.5–100.0] (65/65)	71.4 [58.4–81.7] (25/35)	86.7 [76.8–93.4] (65/75)	100.0 [86.3–100.0] (25/25)	85.7 [77.3–91.9]

*SCT* standard computed tomography, *DECT* dual-energy computed tomography, *MRI* magnetic resonance imaging, *AUC* area under the curve, *PPV* positive predictive value, *NPV* negative predictive value

^†^ Data expressed as percentages, with raw data in parentheses and 95% CIs in brackets

The three readers independently scored the SCT, DECT, and MRI images for diagnostic confidence (Table 3). DECT images were rated from good to excellent for visualizing both non-color-coded and color-coded supraspinatus tendons. There was no significant difference in diagnostic confidence between uncolored and color-encoded supraspinatus tendons (*p* > 0.05).

**Table 3:** The diagnostic confidence scores of SCT, DECT (mono+ 50 keV, mono+ 90 keV), and MRI for the visualization of the supraspinatus tendon injury

	STT	No STT
SCT		
Scores^*^	3.4	3.9
Mono+ 50 keV†		
Scores^*^	4.0	4.5
Mono+ 50 keV^†^		
Scores^*^	4.2	4.7
Mono+ 90 keV†		
Scores^*^	4.0	4.7
Mono+ 90 keV^†^		
Scores^*^	4.3	4.9
MRI		
Scores^*^	5.0	5.0

*STT* supraspinatus tendon tear

^*^ Data expressed as the mean value of readers

† Uncolor-coded for the visualization of the supraspinatus tendon

^†^ Color-coded for the visualization of the supraspinatus tendon

### Quantitative analysis of tear, degeneration, and normal areas

**Fig. 6:**
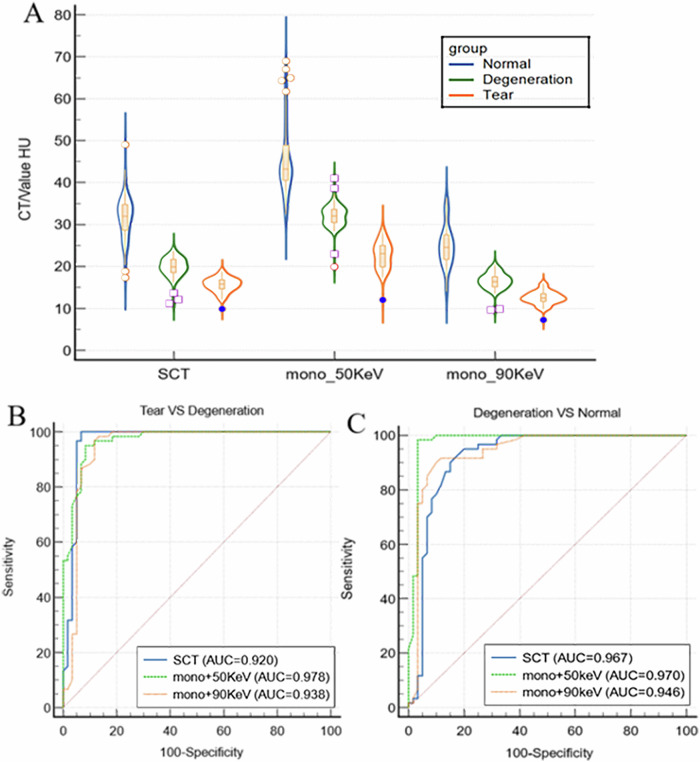
Data distributions of tear, degeneration, and normal areas on SCT, mono+ 50 keV and mono+ 90 KeV images (**A**). Using MRI as the reference standard, the CT attenuation values for torn supraspinatus tendons on SCT, mono+ 50 keV, and mono+ 90 keV images were significantly lower than those in degeneration and normal areas (*p* < 0.001). ROC curves of the SCT, mono+ 50 keV, and mono+ 90KeV CT attenuation values in differentiating torn and degenerative rotator cuff (**B**), and in differentiating degenerative and normal rotator cuff (**C**). CT, computed tomography; ROC, receiver operating characteristic; SCT, standard computed tomography; MRI, magnetic resonance imaging

Using MRI images as the reference standard, the CT attenuation values of torn supraspinatus tendons on SCT, mono+ 50 keV, and mono+ 90 keV images were significantly lower than those of the degenerated areas (*p* < 0.001) (Fig. 6). The AUCs for CT attenuation values in SCT, mono+ 50 keV, and mono+ 90 keV images were 92.0% (optimal cut-off value = 17.4 HU, *p* < 0.001), 97.8% (optimal cut-off value = 28.0 HU, *p* < 0.001), and 93.8% (optimal cut-off value = 14.2 HU, *p* < 0.001), respectively (Fig. 6 and Table 4).

**Table 4:** The CT attenuation values reveal degeneration, and normal areas on SCT, mono+ 50 keV and mono+ 90 KeV images

	SCT value, HU	50 keV value, HU	90 keV value, HU	*p* value
Tear	15.6 ± 1.8	22.6 ± 3.3	12.5 ± 1.6	< 0.001
Degeneration	19.9 (18.5–22.7)^⁎^	32.0 (30.5–36.7)^⁎^	16.4 (15.1–19.6)^⁎^	< 0.001
Normal	31.6 ± 5.5^†^^,^^⁎^	43.2 (40.5–64.7)^†,⁎^	24.9 ± 5.0^†,⁎^	< 0.001
Tear vs degeneration				
Optimal cut-off value	17.4	28.0	14.2	
AUC (90% CI)	0.920 (0.856–0.962)	0.978 (0.933–0.996)	0.938 (0.879–0.974)	> 0.05
Degeneration vs normal				
Optimal cut-off value	23.8	36.1	19.6	
AUC (90% CI)	0.967 (0.917–0.991)	0.970 (0.921–0.992)	0.946 (0.890–0.979)	> 0.05

Data expressed as mean ± standard deviation

Data expressed as median (p25–p75)

*SCT* standard computed tomography, *HU* Hounsfield units, *AUC* area under the curve

^⁎^ Value was statistically different from that of the tear group

^†^ Value was statistically different from those in the degeneration group

A *p* value < 0.05 indicated statistical significance and was highlighted in boldface

The CT attenuation values of the degenerated supraspinatus tendons in the SCT, mono+ 50 keV, and mono+ 90 keV images were significantly lower than those of the normal areas (*p* < 0.001) (Fig. 6). The AUCs for CT attenuation values in SCT, mono+ 50 keV, and mono+ 90 keV images were 96.7% (optimal cut-off value = 23.8 HU, *p* < 0.001), 97.0% (optimal cut-off value = 36.1 HU, *p* < 0.001), and 94.6% (optimal cut-off value = 19.6 HU, *p* < 0.001), respectively (Fig. 6 and Table 4). The measurements demonstrated excellent intrarater and interobserver reliability (κ = 0.65, 0.76, 0.66).

## Discussion

Imaging is vital for diagnosing rotator cuff injuries. In this study, we qualitatively and quantitatively evaluated supraspinatus tendon injuries using DECT. The results indicated that DECT-VMI enhanced the diagnostic accuracy and reliability for detecting supraspinatus tendon tears. Moreover, the CT attenuation values of tears and degeneration in the SCT, mono+ 50 keV, and mono+ 90 keV images were significantly lower than those of normal tendon regions (*p* < 0.001). This underscores the potential of DECT for imaging supraspinatus tendon injuries.

DECT-VMI is generated using single-source instant-switch kVp technology, where the high-voltage generator switches between 80 kVp and 140 kVp within milliseconds to analyze the projection data space [9]. Different tissues exhibit distinct attenuation characteristics as the X-ray energy changes [10]. Low monoenergetic levels can increase the density resolution and imaging contrast resolution. Lyu et al [11] observed that low-keV VMI offers similar or higher signal-to-noise ratios, lesion detectability, and overall image quality perception. The authors also observed that 50 keV VMI excelled in detecting small liver metastases. Higher monoenergetic levels reduce the image contrast but minimize metal artifacts. Winklhofer et al [12] found that DECT monoenergetic imaging outperformed multimaterial and conventional mixed imaging by reducing radiation-hardening artifacts around the pelvis. In detecting rotator cuff injuries, our study found statistically significant differences between SCT and DECT (mono+ 50 keV and mono+ 90 keV) images for diagnosing supraspinatus tendon tears. Possible reasons for these differences include the enhanced soft tissue contrast revealed by DECT, particularly at lower energy levels (50 keV), which improves differentiation between tendons and surrounding tissues. In addition, the ability of DECT to reduce bone-related artifacts at higher energy levels (90 keV) further enhances the visibility of soft tissue structures such as the supraspinatus tendon. Additionally, monoenergetic imaging optimizes image quality by minimizing beam hardening and noise, allowing for better detection of subtle structural changes such as tears or fluid accumulation around the tendon [13–15]. The combination of these factors likely contributes to the superior sensitivity DECT has for detecting supraspinatus tendon tears.

Using shoulder arthroscopy as the gold standard, we found that DECT images demonstrated significantly better accuracy compared to SECT and were not significantly lower than MRI in detecting supraspinatus tendon tears. DECT scans are quick to complete, and flexible post-processing methods, such as MPR reconstruction at any angle, provide enhanced visualization of supraspinatus tendon tears [16]. Therefore, when MRI is contraindicated or unavailable, DECT can serve as a valuable alternative or adjunct tool, offering clinicians a broader diagnostic range. Additionally, our study supports radiologists in confidently diagnosing supraspinatus tears when a lower density zone is observed within the tendon on DECT. We also assessed both uncolored and color-encoded images and found that color-encoded images did not improve the radiologists’ diagnostic confidence, possibly because of increased image noise [17].

Additionally, we performed a quantitative analysis of CT attenuation values in different anatomical regions within the rotator cuff. We found significant differences in CT attenuation values among torn, degenerated, and normal tendon areas on SCT and DECT images. Specifically, the CT attenuation values of the torn regions were significantly lower than those of the degenerated areas, which in turn were lower than those of the normal tendon regions. Liu et al [8] found that DECT can diagnose ACL tears through quantitative methods, identifying an optimal CT cut-off value of 61.8 HU at 80 keV. These quantitative differences emphasize DECT’s ability to discern subtle changes in the tendon and ligament composition, enabling accurate pathological localization and characterization.

Compared with MRI scans, CT scans are readily available, expediting the diagnosis and treatment of acute rotator cuff injuries. Faster diagnosis reduces waiting times, mitigates damage to surrounding structures, and accelerates treatment, ultimately improving patient outcomes and reducing the socioeconomic burden in healthcare and the workplace.

This study had two main advantages. First, DECT-VMI improved the detection rate of supraspinatus tendon tears. Second, the CT attenuation values of the SCT, mono+ 50 keV, and mono+ 90 keV images provide a new quantitative approach for diagnosing rotator cuff injuries. This study also has some limitations. First, regarding qualitative diagnosis, we focused only on supraspinatus tendon tears and did not examine infraspinatus and subscapularis tendon tears. Second, we did not differentiate between partial and full supraspinatus tendon tears. Third, using MRI as the reference standard for the assessment of tendon degeneration was subject to artifacts and variations in image quality. Fourth, our study cohort included patients who were referred for imaging due to suspected rotator cuff pathology, possibly introducing selection bias. Fifth, the study was conducted at a single institution. However, we hope to mitigate this limitation by providing detailed descriptions about our imaging protocols and assessment criteria, allowing for replication and comparison across different institutions. We recommend future multicenter studies to validate our findings across different populations and clinical settings. This will help assess the robustness and generalizability of DECT for evaluating tendon degeneration and rotator cuff injuries.

In conclusion, our findings confirmed the qualitative and quantitative diagnostic capabilities of DECT for detecting supraspinatus tendon injuries with excellent accuracy and reliability. DECT VMI enhanced the visualization of supraspinatus tendon tears. Its quantitative capabilities demonstrated its potential for assessing supraspinatus tendon pathology in clinical settings. Further research is needed to verify these findings and fully explore the potential of DECT, particularly concerning its utility in postoperative follow-up evaluations of the rotator cuff.

## Supplementary information

ELECTRONIC SUPPLEMENTARY MATERIAL

## Abbreviations

AUC: Area under the curve
DECT: Dual-energy computed tomography
FOV: Field of view
HU: Hounsfield units
MRI: Magnetic resonance imaging
NPV: Negative predictive value
PPV: Positive predictive value
SCT: Standard computed tomography
VMI: Virtual monochromatic imaging
