# Correction: Diagnostic value of dual-energy CT virtual monochromatic imaging for supraspinatus tendon injuries: a comparison with standard CT and MRI

**DOI:** 10.1007/s00330-025-12130-x

**Published:** 2025-11-03

**Authors:** Suwei Liu, Qizheng Wang, Yali Li, Ming Ni, Chenyu Jiang, Wenhuan Li, Huishu Yuan

**Affiliations:** 1 Department of Radiology, Peking University Third Hospital, Beijing, China; 2 CT Research Center, GE Healthcare China, Beijing, China

**Correction to: European Radiology**

10.1007/s00330-025-11760-5 published online 14 June 2025.

The authors discovered a typo error in the “Results” section of the article. Specifically, the sentence:

“SCT and DECT showed 16 false positives (16 out of 43 negative cases) and 7 false positives (7 out of 34 negative cases), respectively. In contrast, the MRI modality showed no false positives: 0 false positives out of 25 negative cases.” contains an incorrect term. The correct sentence should read:

“SCT and DECT showed 16 false negatives (16 out of 43 negative cases) and 7 false negatives (7 out of 34 negative cases), respectively. In contrast, the MRI modality showed no false negatives: 0 out of 25 negative cases.” should have stated “false negatives” instead of “false positives” in this sentence. We inadvertently wrote “false positives,” which understandably led to confusion.

This mistake may lead to a misunderstanding of the diagnostic performance. The correction does not affect the conclusions of the study.

The original article has been corrected.

