# Supplementary material for: Diagnostic value of dual-energy CT virtual monochromatic imaging for supraspinatus tendon injuries: a comparison with standard CT and MRI
Source: Eur Radiol. 2025 Jun 14;35(12):7877–87. doi: 10.1007/s00330-025-11760-5 (PMC12634753; doi:10.1007/s00330-025-11760-5)
Supplement: Supplementary file 1 — ELECTRONIC SUPPLEMENTARY MATERIAL [file 330_2025_11760_MOESM1_ESM.pdf]

Diagnostic value of Dual-energy CT Virtual Monochromatic Imaging for Supraspinatus Tendon Injuries: A comparison with standard CT and MRI

ELECTRONIC SUPPLEMENTARY MATERIAL

parameters	Ocor T1WI FSE	Tra PDWI FSE	OCor PDWI FSE	OSag PDWI FSE
Repetition time,ms	510	3000	3110	3110
Echo time,ms	16	80/33	65	65
Echo train length	6	6	6	6
No.excitations	2	2	2	2
Bend width,Hz	220	220	220	200
Flip angle,degrees	150	150	90	90
Field of view,mm	160×160	160×160	160×160	160×160
Matrix	256×256	256×256	240×320	240×320
Slice thickness/gap,mm	3.0/0.3	3.0/0.3	3.0/0.3	3.0/0.3
Mean acquisition time,min:s	2:44	3:32	3:22	3:28

Table S1: MRI scan acquisition

Note: Ocor =oblique coronary position, Osag= oblique sagittal position, Tra= transverse position, T1WI= T1 weighted imaging, PDWI= proton density weighted imaging, FSE= Fast Spin Echo.

Table S2: Process for determining the optimal VMI and slice thickness.

	Parameters	40 kev	50 kev	60 kev	70 kev	80 kev	90 kev	100 kev	110 kev	120 kev	130 kev	140 kev
1mm	Image noise	3.0	3.2	3.2	3.2	3.3	3.4	3.4	3.5	3.6	3.6	3.8
	Lesion prominence	3.7	3.6	3.5	3.5	3.5	3.5	3.4	3.2	3.2	2.5	2.0
	Image quality	2.8	3.0	3.0	3.1	3.1	3.2	3.2	3.3	3.4	3.5	3.6
	Diagnostic confidence	3.5	3.6	3.6	3.6	3.5	3.5	3.4	3.2	3.0	2.6	2.0
	Overall score	13.0	13.4	13.3	13.4	13.4	13.6	13.4	13.2	13.2	12.2	11.4
2mm	Image noise	3.6	3.8	3.8	3.9	3.9	4.0	4.0	4.2	4.2	4.3	4.4
	Lesion prominence	4.0	4.0	3.8	3.8	3.8	3.8	3.5	3.4	3.0	2.5	2.4
	Image quality	3.6	3.8	3.8	3.9	3.9	4.0	4.0	4.0	4.2	4.2	4.2
	Diagnostic confidence	3.9	4.0	4.0	3.8	3.9	3.9	3.7	3.6	3.5	3.0	3.0
	Overall score	15.1	15.6	15.4	15.4	15.5	15.7	15.2	15.2	14.9	14.0	14.0
3mm	Image noise	4.4	4.5	4.5	4.5	4.5	4.6	4.6	4.7	4.7	4.8	4.8
	Lesion prominence	4.7	4.7	4.5	4.5	4.5	4.5	4.2	4.0	3.0	2.8	2.5
	Image quality	4.5	4.6	4.6	4.6	4.7	4.8	4.8	4.8	4.8	4.8	4.8
	Diagnostic confidence	4.5	4.8	4.7	4.7	4.6	4.6	4.4	4.0	3.8	3.5	3.0
	Overall score	18.1	18.6	18.3	18.3	18.3	18.5	18.0	17.5	17.5	15.9	15.1

Note: Data expressed as mean value of readers. Interreader agreement was evaluated by computing weighted Fleiss k. Interreader agreement was good for reader1 as well as for reader2 (k = 0.66).
